# Training doctors to manage patients with multimorbidity: a systematic review

**DOI:** 10.15256/joc.2016.6.87

**Published:** 2016-08-26

**Authors:** Cliona Lewis, Emma Wallace, Lorraine Kyne, Walter Cullen, Susan M. Smith

**Affiliations:** ^1^Department of General Practice, Royal College of Surgeons in Ireland Medical School, Dublin, Ireland; ^2^HRB Centre for Primary Care Research, Department of General Practice, Royal College of Surgeons in Ireland Medical School, Dublin, Ireland; ^3^Department of Medicine for the Older Person, Mater Misericordiae University Hospital, Dublin, Ireland; ^4^School of Medicine, University College Dublin, Dublin, Ireland

**Keywords:** Multimorbidity, postgraduate training, patient management, postgraduate education

## Abstract

**Background:**

Patients with multimorbidity (two or more chronic conditions) are now the norm in clinical practice, and place an increasing burden on the healthcare system. Management of these patients is challenging, and requires doctors who are skilled in the complexity of multiple chronic diseases.

**Objective:**

To perform a systematic review of the literature to ascertain whether there are education and training formats which have been used to train postgraduate medical doctors in the management of patients with multimorbidity in primary and/or secondary care, and which have been shown to improve knowledge, skills, attitudes, and/or patient outcomes.

**Methods:**

Overall, 75,110 citations were screened, of which 65 full-text articles were then independently assessed for eligibility by two reviewers, and two studies met the inclusion criteria for the review.

**Results:**

The two included studies implemented and evaluated multimorbidity workshops, and highlight the need for further research addressing the learning needs of doctors tasked with managing patients with multimorbidity in their daily practice.

**Conclusion:**

While much has been published about the challenges presented to medical staff by patients with multimorbidity, published research regarding education of doctors to manage these problems is lacking. Further research is required to determine whether there is a need for, or benefit from, specific training for doctors to manage patients with multimorbidity.

PROSPERO registration number: CRD42013004010.

## Introduction

Multimorbidity can be defined as the co-existence of two or more chronic conditions in an individual [1] where one of these conditions is not necessarily more central than the other(s) [2]. Patients with multimorbidity are now the norm in clinical practice, with prevalence ranging from 13 to 72%, depending on the methodology used and the setting [3]. Factors such as social deprivation, psychiatric illness, and coexisting intellectual disability are associated with an increased prevalence of multimorbidity [4, 5].

While much attention has been directed at the management of chronic disease, it is the multiplicity of disease rather than the chronicity that increases demands on healthcare systems [6]. As the prevalence of multimorbidity increases, its impact on both the healthcare system and the people using that system depends, in part, on the competence of doctors who treat patients with multiple illnesses [7]. Management of patients with multimorbidity has been the focus of a recent British Medical Journal Clinical Review [8], and the National Institute for Health and Care Excellence (NICE) has published draft guidance on this topic in April 2016 with full guidance due later in Autumn 2016 [9]. These developments address the limitations of current clinical guidelines which are predominantly based on single-disease–focused research and present recommendations that may be inappropriate when applied to patients with multimorbidity [10–12].

Critical in the appropriate management of patients with multimorbidity is the doctor–patient consultation, which must take into account the need for extensive information-gathering and record-keeping, the changing priorities over time, and the need for high-quality communication to coordinate care with other services and healthcare providers [13]. The increasing complexity of disease combinations presenting to medical professionals requires additional skills and training so that clinicians caring for patients with multimorbidity can competently and confidently manage the multiple chronic diseases presented, and implement a personal, patient-centred approach to care, involving shared decision-making, patient and carer education, and self-management [14–16]. The management of older patients with multimorbidity and frailty is embedded in higher specialist training in Geriatric Medicine in Ireland and the UK [17, 18]. The growing ageing population, limited numbers of trained geriatricians, and increasing numbers of patients with multimorbidity is a challenge, and should generate increased training of ‘generalists’, ideally within family practice, with a view to facilitating the maintenance of these patients in the community as much possible [19]. Management of polypharmacy, lack of guidelines and decision-making tools, and the difficulty of trying to manage multiple problems in a single, fixed-time consultation are just some of the challenges described by doctors in the qualitative literature examining doctors’ views on multimorbidity [20–23]. As doctors feel inadequately trained in these and other compentencies which are critical to the management of patients with multimorbidity, sufficient, comprehensive and validated training must be provided to optimize patient outcomes in people with this increasingly ‘normal’ presentation [24].

This systematic review aimed to ascertain whether there are education and training formats which have been used to train postgraduate medical doctors in the management of patients with multimorbidity in primary and/or secondary care, and which have been shown to improve knowledge, skills, attitudes, and/or patient outcomes.

## Methods

### Study design

A systematic review of the literature was performed and reported according to the Preferred Reporting Items for Systematic Reviews and Meta-Analyses (PRISMA) standardized reporting guidelines [25].

### Criteria for considering studies for this review

As no previous reviews had been conducted in this area, we aimed to identify all published evidence relating to this topic, and included articles of any type which addressed postgraduate medical education and training in the management of patients with multimorbidity in primary or secondary care. 

Studies were eligible for inclusion if they recruited graduate medical doctors who had participated in a training programme addressing management of patients with multimorbidity in primary or secondary care. All educational and training formats were included, and both observational and experimental study designs were eligible. Studies were excluded if they addressed only clinical management or organizational interventions for patients with multimorbidity, or if they related only to either undergraduate training or training for health professionals other than doctors.

### Outcomes

Primary outcomes were any measure of doctor knowledge, attitude, or skills that related to the content of the training programme. Secondary outcomes included any patient outcomes reported in a study that examined an intervention designed to train doctors to manage multimorbidity including patient-reported outcome measures, for example, health-related quality of life and health-service utilization in patients with multimorbidity.

### Search strategy

Initial scoping searches in late 2012 suggested that there was very little published literature regarding multimorbidity and education, so our search was widened to include editorials, news pieces, and commentaries in an effort to maximize yield of relevant papers. The principal challenge of this search was the fact that there is currently no Medical Subject Heading (MeSH) term for multimorbidity. A search string was initially developed using keywords to capture the concept of multimorbidity, based on previous published searches [26, 27].

Systematic literature searches were initially conducted in April 2013 and updated regularly up to January 2016. We searched databases from 1996 onwards, as the concept of multimorbidity was first defined in 1998 by Van den Akker *et al.* [1]. The search databases and search strategy are provided in the Supplementary Methods. We also hand-searched the reference lists of included articles and other articles of interest. We contacted authors involved in the field, those who had published related or pilot work, and searched the International Research Community on Multimorbidity archive [28]. We did not exclude papers on the basis of language.

### Data collection and analysis

#### Study selection

One author (C.L.) screened the titles and abstracts, and full-text copies of potentially relevant papers were obtained for further evaluation. These were independently assessed for eligibility by at least two reviewers (C.L. and either S.S. or E.W.) and the final included studies were confirmed as eligible by three authors (C.L., S.S., E.W.).

#### Data extraction and management

Two review authors independently extracted data from each included paper, using a data extraction form specifically designed for this study. Data extracted included study design, setting and definition of multimorbidity, intervention, characteristics of participating providers of intervention, characteristics of participating doctors (being trained), quality criteria, source of funding, ethical approval, outcome measures, and length of post-intervention follow-up period.

Disagreements were resolved by discussion and consensus.

#### Assessment of risk of bias in included studies

Risk of bias of the included studies was assessed using the Cochrane Collaboration Risk Of Bias In Non-randomized Studies of Interventions (ROBINS-I) assessment tool [29]. The domains assessed are presented in Table 1.

#### Data analysis

We anticipated that meta-analysis would not be possible and planned to conduct a narrative synthesis of included studies.

## Results

### Search results

**Figure 1:**
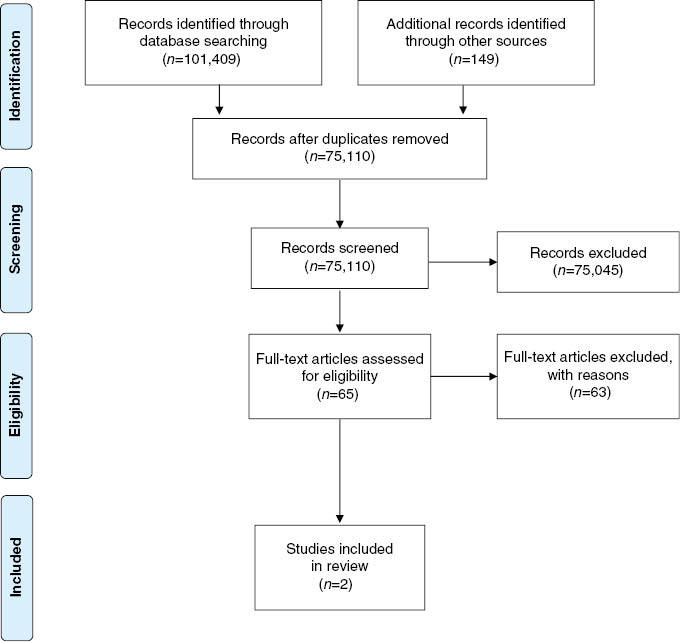
PRISMA flow diagram.

Overall, 75,110 citations were screened, of which 65 full texts were deemed to be potentially relevant. These 65 articles were formally independently assessed for eligibility by two reviewers. Two studies met the inclusion criteria for the review, as outlined in the flow diagram (see Figure 1). Excluded studies and reasons for exclusion can be found in Supplementary Table 1.

### Characteristics of included studies

Two studies met the inclusion criteria and are summarized in Table 2. Both studies had non-randomized controlled trial designs with one (Andolsek *et al*. [30]) being a non-randomized controlled study and the other (Maguire *et al*. [31]) being an uncontrolled before and after study, described as a pilot study.

As summarized in Table 2, Andolsek *et al*. reported on a half-day workshop for family physicians and internal medicine physicians, physician assistants, and nurse practitioners, which addressed complex clinical scenarios [30]. While multimorbidity is not defined in this paper, the cases described had multiple, co-existing chronic conditions.

Andolsek *et al.*’s workshops comprised two parts: a large group presentation during which guidelines, algorithms, and clinical evidence were summarized by primary care faculty; followed by small group discussions about developing plans for the diagnosis and management of a number of complex case scenarios [30]. Their intervention group of 487 practitioners contained 307 doctors, while their control group comprised 992 participants, 605 of whom were doctors. The clinical cases used in the workshop related to aspects of multimorbidity care that are recognized to be challenging; including patient factors such as self-care, lifestyle change, and medication concordance; and health profession issues, including care coordination. The control group in this study did not attend the live workshop, but completed a complex cases module online which incorporated the content of the workshop and evidence-based strategies for management of patients with multimorbidity. The effectiveness of the online module was measured using the same questionnaire that evaluated the workshop, administered both before and immediately after each online case study [30].

The intervention in Maguire *et al.*’s uncontrolled before–after study [31] was described as a 2-hr multimorbidity workshop for 20 postgraduate trainees in General Practice, in the northwest of Ireland. The workshop was facilitated by the directors of the general practitioner (GP) training scheme, who assessed recall of prior knowledge via a questionnaire at the beginning of the workshop. The trainers then presented a multimorbidity literature review to the trainees, before facilitating small group discussion of ‘simulated multimorbidity cases’. These simulated multimorbidity cases were developed by the facilitators and were based on clinical cases that they had encountered in practice. Each case involved information about a year of the patient’s care, challenges for both the doctor and the patient, and the social history of the patient. A plenary talk at the end of the small group work summarized the proceedings, and the workshop closed with a knowledge questionnaire and an evaluation by the trainees of the workshop content [31].

### Intervention development

The clinical topics included in the intervention developed by Andolsek *et al*. were based on an ‘independent educational needs assessment conducted by DukeCME and the accredited continuing medical education (CME) provider’ [30]. Andolsek *et al.* suggest that realistic, occupationally appropriate settings, with an opportunity to discuss the cases with colleagues, should be used to deliver novel clinical information, referencing a paper by Moore *et al*. [32]. While this is not specific to multimorbidity, they suggest that presenting information in a discursive format, in an authentic work setting, facilitates the implementation of new clinical information into practice [32].

Description of the theoretical basis of the development of the pilot workshop by Maguire *et al*. was not reported and they suggest that a needs assessment is necessary for future workshops: given that it is a pilot project, this may follow when subsequent work is published. Facilitators of the workshop based the included cases on prior patient contacts, and trainees were given information about a patient’s medical and social history, along with available relevant guidelines [31].

### Outcome assessment

As outlined in Table 2, Andolsek *et al*. evaluated their workshop with both an immediate satisfaction questionnaire and two non-validated, follow-up surveys which were completed at least 30 days after the workshop, and were developed by the authors [30]. Each follow-up survey included single-best-answer questions about three complex cases, and was administered to each participant to assess clinical knowledge. Participants were also asked about their confidence in managing patients with multiple comorbidities as well as the significance of barriers to treating these patients. The control participants completed a complex cases module online, and its effectiveness was measured by the same questions described above both before, and immediately after each case study contained in the online module [30].

In the other included study, GP trainees attending Maguire *et al.*’s pilot workshop completed a pre- and post-workshop knowledge questionnaire that was developed by the investigators, details of which are not included in the publication [31]. As such, direct comparison of the two outcome measurement tools is not possible in the context of this review.

### Effectiveness of educational interventions

Both studies reported non-validated measures of doctor knowledge and skill assessed on completion of the training [30, 31]. Andolsek *et al.* reported that the majority of the intervention participants (physicians and non-physicians) described an increase in their knowledge (96%) and self-reported competence (89%) on immediate completion of the workshop [30]. Thirty days following workshop completion, surveys were sent to 247 of the 307 physician participants and of these, 62 (25%) responded. Those who responded self-reported that knowledge had increased in a number of areas that were addressed at the workshop: two of eight specific areas reported were significantly improved when compared with non-participant controls (recognition of medications that contribute to an overactive bladder, and appropriate referral of patients with rheumatoid arthritis to specialty care). There was no difference in self-reported confidence related to treatment decisions. The authors state that doctors who participated in the workshop reported that they were 27% more likely than non-participants to use available evidence and guidelines in practice: data to support this are not provided. Significant gains in knowledge were seen in almost all (17/18) assessment areas for the 992 clinicians who completed the online cases (survey response rates are not provided). No long-term follow-up of the online case participants was reported, so it is not possible to compare the online and workshop modalities at that point in follow-up [30].

GP trainees who attended Maguire *et al.’s* pilot workshop were found to have improved knowledge of the characteristics of multimorbidity (80% after the workshop compared with 25% before the workshop) [31]. All 20 trainees reported improved understanding and increased confidence in the management of patients with multimorbidity in the community. Neither study reported any of the secondary outcomes outlined in the review protocol, nor did they evaluate the long-term impact of the training which was provided [30, 31].

### Risk of bias in included studies

The two included studies were assessed using the ROBINS-I tool, and both were found to be at high risk of bias [29–31] (Table 1). Confounding was a serious risk in both studies due to the study designs, and selection of participants was also deemed to be at high risk of bias. Missing data are not reported by Maguire *et al.* [31], but there was a serious risk of bias in Andolsek *et al.*’s study due to low response rates (20%) to questionnaires 30 days after the workshop [30]. Both studies used non-validated, subjective outcomes, and neither reported blinding of outcomes assessment [30, 31]. As such, the overall risk of bias in the two studies included in this review is high.

## Discussion

### Summary of findings

This systematic review identified only two studies that developed and evaluated training programmes for doctors in managing patients with multimorbidity [30, 31], despite an extensive search over several years. The evidence determining the effectiveness of multimorbidity educational interventions for doctors is very limited, and the paucity of studies addressing this topic was surprising. The two included studies indicate that it is feasible to deliver workshop or online training over a short period of time to physicians on this topic. The effectiveness of these programmes has yet to be confirmed, but the study by Maguire *et al.* was a pilot programme, and could be rolled out and subsequently evaluated [31]. The programme by Andolesk *et al.* appears to favour a workshop format over online case module [30], although a more robust evaluation of the two formats is required.

### Comparison with existing literature

This is a challenging area: patients with multimorbidity are a heterogeneous group. While some disease combinations are common, many permutations exist, each with individual requirements, therapeutic strategies, and targets. As to why there is so little published about training of doctors in this area, it is likely that since the concept of multimorbidity is a relatively recent one, it is partly a function of time: the focus of investigators in the area in recent years has been on therapeutic strategies and guideline development. It inevitably takes time for the educational arm to emerge, particularly where there is still such uncertainty as to how best to manage patients with multimorbidity. While some research has been conducted in this area with respect to training of undergraduates, we have been unable to identify any systematic reviews of the effectiveness of related postgraduate educational interventions, such as training in the management of complexity in clinical practice [33]. While the postgraduate curricula may have changed, we have not seen a corresponding increase in published literature regarding specific training in multimorbidity: perhaps the training is integrated into existing modules, or is indeed a ‘re-naming’ of already delivered material, and as such, not considered novel to the trainers.

### Strengths and weaknesses of the review

To our knowledge, this is the first study to systematically review the literature focusing on postgraduate training of medical doctors in the area of multimorbidity. The search was broad and inclusive, but the findings need to be interpreted in the context of some limitations.

The relatively recent introduction of the term ‘multimorbidity’, its lack of definition, and the current absence of a MeSH term proved a significant challenge when developing the search string, giving a large number of search results to be screened [34]. It is possible that there are relevant publications that were omitted as a result; however, multiple searches were conducted and international experts in multimorbidity were contacted.

### Implications for future research

There is clearly huge scope for future research in this area. Initial assessment of learning needs is vital to enable educators to provide doctors with relevant and practical training to address the clinical challenges presented by patients with multimorbidity. Just as with treatment of patients, medical training tends to focus on individual diseases: further research is required to delineate deficiencies in the current curricula, so that focused training can be provided.

The studies included in this review examine case-based interventions using workshop and on-line formats [30, 31]. However, learning styles differ, and it is unlikely that a ‘one size fits all’ educational format will accommodate all preferences [35]; rather, several educational modalities will be needed in the CME scenario, covering the same material, but either online, in a workshop, in print, or other modality. Further research should be directed at determining the preference of doctors with regard to various formats of delivery and the differential effectiveness of each format. Outcomes (both doctor- and patient-related) should be clearly defined and ideally blindly assessed: there was a disappointing absence of measurement of change in patient outcomes or change in practice. In order to extract maximum gain from any multimorbidity training programme or module, it is important that it is robustly designed and thoroughly evaluated to enhance participants’ clinical practice.

### Implications for policy and practice

Although there is broad recognition that patients with multimorbidity require care to be delivered by trained generalists rather than single-system specialists [36, 37], there remains a shortage of generalists in many countries. Providing more training positions in General Practice, General Internal Medicine and Geriatric Medicine should improve the ability of the health system to manage these complex patients in appropriate settings, both within the community and on an in-patient basis, if needed. Provision of more generalists alone will not suffice, and the available qualitative literature suggests that GPs feel underprepared to manage these patients [20, 22].

**Figure 2:**
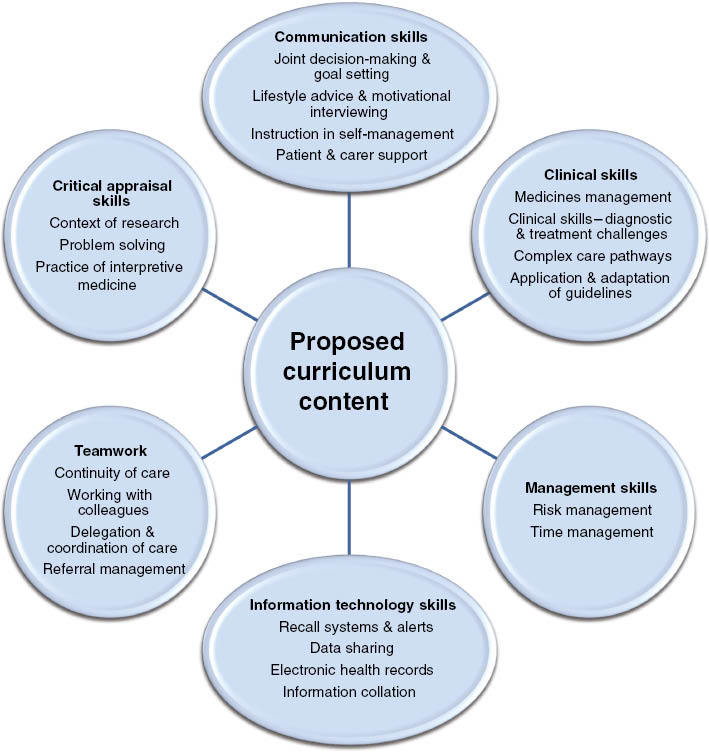
Proposed curriculum content for training of doctors in management of patients with multimorbidity [7, 20–22, 39–45].

While there is limited evidence to support clinical practice management of patients with multimorbidity, the findings from this review can be considered along with the existing qualitative literature on doctors’ views, two recent clinical review papers providing guidance to doctors managing patients with multimorbidity and the recently published draft NICE Clinical Guidelines on Multimorbidity [8, 9, 21–23, 38]. This and other literature highlight a range of areas that need to be addressed to enable doctors to confidently manage patients with multiple chronic conditions. Figure 2 synthesizes some of the topics suggested by the published literature, and might provide a basis for curriculum development for multimorbidity education [7, 20–22, 39–45].

Postgraduate medical educators need to consider who is best suited to training doctors who manage patients with multimorbidity. Management of these patients is complex, and the two studies included in this review used case-based approaches delivered by medical doctors. This is supported by both Knowles’s adult learning theory [46] and Kolb’s model of experiential learning [47], in which concrete experience is followed by reflection, abstract conceptualization, and subsequent active experimentation. However, there is also a role for other healthcare professionals in training: the proposed curriculum components outlined in Figure 2 suggest that a range of other disciplines, such as communication specialists, simulated patients, and other healthcare workers, may have a role to play in training doctors: for example, pharmacists may have a key role in supporting training in medicines management.

Additional consideration needs to be given to when doctors should be trained. Given the prevalence of multimorbidity in the community, some educators suggest that training in its management should begin at the undergraduate level [48]. Training should certainly be integrated into postgraduate medical training, ideally for doctors of all specialties, to enable competent basic management of multimorbidity by doctors of all medical and surgical specialties. For doctors who have completed their specialist or generalist training, updates could be incorporated into CME training, with regulatory authorities advising on frequency of training and updates.

Optimal educational format is a significant issue which needs further research: while the two studies included in our review both implemented and evaluated workshops, they are clearly not the only format available to train doctors to manage patients with multimorbidity. Andolsek *et al*. did not find significant differences in outcomes when workshop training was compared with an online learning module completed by their control group [30]. Given the diversity of doctors to be trained, and the importance of training in this area to be an ongoing, realistic learning experience, updated over time in a CME scenario, it is unlikely that a single-delivery format will suit all participants. This may present an opportunity to utilize distance learning or remote learning modules. However, the preferred format for doctors with regard to learning in this area has yet to be determined, and needs to be explored prior to development and implementation of training. Given the demands on time and finances of doctors, we suggest that any training on the management of multimorbidity in practice must be practical, needs-driven, stimulating, evidence-based, longitudinal, and outcome-oriented, in order to change practice and ideally improve clinical outcomes for complex patients.

## Conclusion

Much has been published about the challenges presented by patients with multimorbidity, but the issue of educating doctors to manage these problems has been poorly addressed. The two studies presented in this review implemented and evaluated multimorbidity workshops, and provide a basis for further research. It remains to be determined whether there is a specific need for training of doctors to manage patients with multimorbidity, and if so, how that need can best be met. It also remains to be proven that improving knowledge, skills, and confidence of doctors results in improved care of this patient group. We have identified existing literature that provides both a platform for training [30, 31], and a basis for curriculum development for training doctors in the management of patients with multimorbidity [7, 20–22, 39–45]. Incorporation of emerging guidelines and research findings into multimorbidity training curricula for doctors with appropriate evaluation of its effectiveness is needed, to change practice and enhance the competence and confidence of doctors in managing this challenging population of patients, with the ultimate aim of improving clinical outcomes.

## Figures and Tables

**Table 1:**
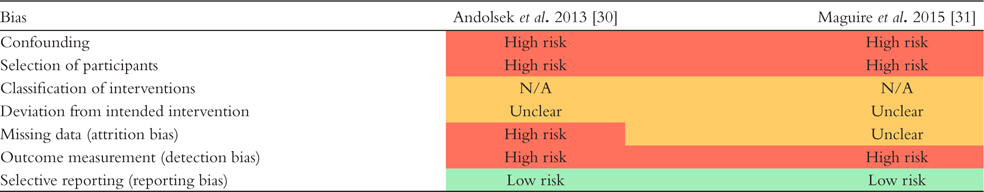
Risk of bias assessment.

Risk of bias assessment was performed using the ROBINS-I tool [29]. N/A, Not applicable.

**Table 2:** Characteristics of included studies.

Study Country Design	Participants	Intervention and comparison	Outcomes
Andolesk *et al*. 2013 [30] USA Non-randomized, controlled trial	1479 Participants: 487 workshop participants and 992 controls Physicians and non-physicians (63% of workshop participants were doctors, 61% of controls were doctors)	*Intervention (workshop)* Large group presentation, reviewing and discussing clinical evidence, current practice guidelines, and available treatment algorithms Small group discussion about challenging case studies, developing diagnostic, and treatment plans *Controls* Completed online case studies of patients with chronic diseases, based on the workshop that was delivered	Immediate post-workshop satisfaction questionnaire Thirty days after workshop: self-reported knowledge, competence, confidence gains, and knowledge related to clinical cases Controls completed knowledge- and competence-based assessment questions before and immediately after each case study
Maguire *et al*. 2015 [31] Ireland Uncontrolled before and after study (pilot study)	20 GP trainees from 4 years of training – some completing hospital jobs, some GP registrars	*Pilot multimorbidity workshop* Presentation of literature review followed by large group discussion Small group work facilitated by programme directors, discussing simulated multimorbidity cases No comparison group	Post-workshop knowledge questionnaire

GP, General practitioner.
